# High-Throughput Screening for Growth Inhibitors Using a Yeast Model of Familial Paraganglioma

**DOI:** 10.1371/journal.pone.0056827

**Published:** 2013-02-22

**Authors:** Irina Bancos, John Paul Bida, Defeng Tian, Mary Bundrick, Kristen John, Molly Nelson Holte, Yeng F. Her, Debra Evans, Dyana T. Saenz, Eric M. Poeschla, Derek Hook, Gunda Georg, L. James Maher

**Affiliations:** 1 Department of Biochemistry and Molecular Biology, Mayo Clinic College of Medicine, Rochester, Minnesota, United States of America; 2 Mayo Graduate School, Mayo Clinic College of Medicine, Rochester, Minnesota, United States of America; 3 Institute for Therapeutics Discovery and Development, College of Pharmacy, University of Minnesota–Twin Cities, Minneapolis, Minnesota, United States of America; 4 Department of Molecular Medicine, Mayo Clinic College of Medicine, Rochester, Minnesota, United States of America; Florida State University, United States of America

## Abstract

Classical tumor suppressor genes block neoplasia by regulating cell growth and death. A remarkable puzzle is therefore presented by familial paraganglioma (PGL), a neuroendocrine cancer where the tumor suppressor genes encode subunits of succinate dehydrogenase (SDH), an enzyme of the tricarboxylic acid (TCA) cycle of central metabolism. Loss of SDH initiates PGL through mechanisms that remain unclear. Could this metabolic defect provide a novel opportunity for chemotherapy of PGL? We report the results of high throughput screening to identify compounds differentially toxic to SDH mutant cells using a powerful *S. cerevisiae* (yeast) model of PGL. Screening more than 200,000 compounds identifies 12 compounds that are differentially toxic to SDH-mutant yeast. Interestingly, two of the agents, dequalinium and tetraethylthiuram disulfide (disulfiram), are anti-malarials with the latter reported to be a glycolysis inhibitor. We show that four of the additional hits are potent inhibitors of yeast alcohol dehydrogenase. Because alcohol dehydrogenase regenerates NAD^+^ in glycolytic cells that lack TCA cycle function, this result raises the possibility that lactate dehydrogenase, which plays the equivalent role in human cells, might be a target of interest for PGL therapy. We confirm that human cells deficient in SDH are differentially sensitive to a lactate dehydrogenase inhibitor.

## Introduction

### Cancer Focus

Paraganglioma/pheochromocytoma (PGL) is a rare neuroendocrine tumor derived from paraganglia, a diffuse neuroendocrine system present from the pelvic floor to the base of the skull [1]. PGL patients may display catecholamine excess with symptoms including headache, sweating, palpitations, and flushing. PGLs have an incidence near 1∶100,000 in the general population [1], [2] with approximately 50% of cases being explained by mutations in one or more of ten PGL susceptibility genes so far described [3]. The penetrance of familial PGL appears to be greater than 40%, depending on genotype. Some PGLs are initially benign and curable by resection. Malignancy is defined by the appearance of distant metastases, commonly to bone, liver, lung, and lymph nodes [4]. Extra-adrenal pheochromocytomas are estimated to be malignant in 15–50% of cases, depending on subtype [5], [6]. There is currently no effective cure for malignant PGL.

### PGL Genetics and Biochemistry

Remarkably, the genes whose defects predispose to PGL are not typical tumor suppressor genes. Five genes encoding subunits of the succinate dehydrogenase (SDH) complex (SdhA, SdhB, SdhC, and SdhD) [7]–[10] and the enzyme that flavinates SdhA [11], [12] are the leading tumor suppressor genes in familial PGL. Even in tumors that are apparently sporadic (not associated with familiar syndromes) various SDH gene mutations were described in up to 24% of cases [5], [13]. Deletions at the same or closely related loci (11q13 and 11q22–23) are observed in some of these cases [14]. The remaining half of familial PGLs result from inherited mutations in von Hippel-Lindau (VHL) syndrome, multiple endocrine neoplasia type 2 (MEN 2), or neurofibromatosis genes [15], [16].

A broad spectrum of SDH mutations has been reported in familial PGL. Mutations in SDHB and SDHC lead to autosomal dominant inheritance of familial PGL. This pattern has recently been extended as well to SDHA [11]. Mutations in SDHD result in imprinted paternal autosomal dominant inheritance, with new mechanistic models recently proposed [17]. The wide range of mutations in SDH subunit genes identified in familial PGL suggests that loss of function of SDH subunits is the common cause of PGL. Our work focuses on PGL models [18] based on disruption of the *SDHB* gene where mutations commonly cause extra-adrenal metastatic PGL [2], [10], [19].

The succinate dehydrogenase (SDH) complex is ancient and highly conserved. The structure of the porcine complex has been solved by X-ray crystallography [20]. SDH catalyzes the oxidation of succinate to fumarate in the tricarboxylic acid (TCA) cycle, shuttling the extracted electrons to the ubiquinone pool of the electron transport chain. The SDH complex (Complex II) is composed of four small subunits situated in the inner mitochondrial membrane.

Familial PGL is thus particularly remarkable because the causative genetic defects in SDH block the TCA cycle, making PGL the example *par excellence* of the Warburg effect [21]. PGL tumor cells apparently depend only on glycolysis as an inefficient source of ATP. Aerobic glycolysis is common particularly in aggressive tumors [22], though the causative relationship remains unknown. The specificity of SDH loss in PGL has led to the hypothesis that it is succinate accumulation, not just TCA cycle dysfunction, that is pathogenic [18], [23].

### Possible Mechanisms of PGL Tumorigenesis

There are several theories of PGL tumor initiation. SDH mutations have been suggested to result in generation of reactive oxygen species (ROS) by disruption of electron flow and improper reduction of water. Mutagenic ROS could damage nuclear proto-oncogenes or tumor suppressor genes, leading to tumorigenesis [24], [25]. When tested in a yeast model of human PGL, ROS levels were detectably increased, but were not obviously mutagenic [18]. There is substantial evidence favoring a pseudohypoxia model of PGL initiation implicating Hypoxia-inducible factors (HIFs) [26]. HIFs are heterodimeric basic helix-loop-helix transcription factors that include the HIF-1β and oxygen-regulated HIFα subunits (HIF-1α, HIF-2α, HIF-3α). Regulation involves oxygen-dependent HIFα proline hydroxylation by prolylhydroxylase (PHD) enzymes, ubiquitin ligation, and proteasomal degradation of the HIFα subunit under normoxic conditions [27]. HIFα prolylhydroxylation requires oxygen, iron, and 2-ketoglutarate (2KG), and the reaction produces succinate (Su) as a byproduct. In hypoxia, prolylhydroxylation is inhibited, and HIFα accumulates, translocates to the nucleus, and pairs with the HIFβ subunit. Thus, HIFα stability is directly regulated by oxygen. Genes stimulated by HIF include transporters for increased glucose import and angiogenesis.

**Figure 1:**
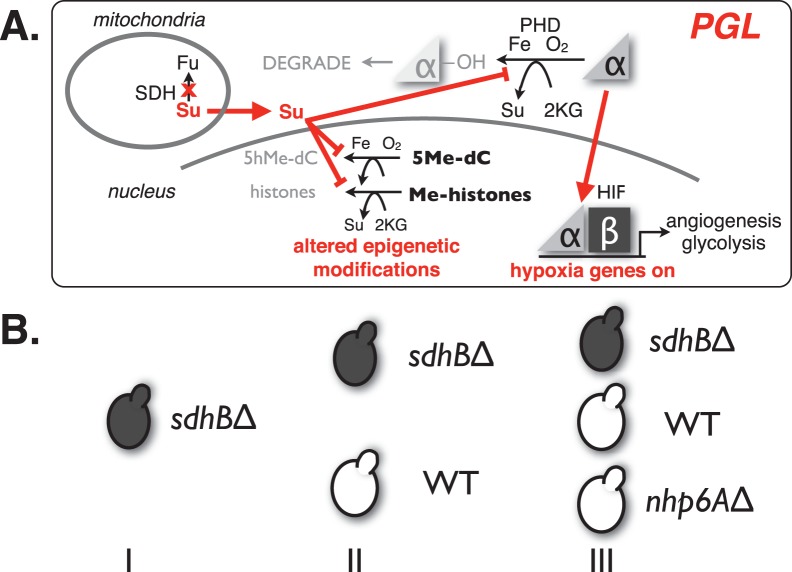
Disease model and HTS strategy. A. Hypothetical molecular basis for PGL tumorigenesis in cells accumulating succinate due to SDH mutations. Putative tumor suppressor functions of Fe/O_2_/2KG-dependent dioxygenases in Hifα degradation and epigenetic regulation of histone methylation and 5-methylcytosine hydroxylation are subject to Su inhibition in PGL. B. A simple PGL tumor model is provided by haploid yeast carrying *sdh2Δ* gene disruption. HTS for compounds differentially toxic to *sdh2Δ* mutant yeast is performed in three stages. I: Identification of compounds inhibitory to the growth of *sdh2Δ* mutant yeast; II: Assessment of differential growth inhibition of WT vs. *sdh2Δ* mutant yeast using repurchased compounds identified in stage I; III: confirmation of differential growth inhibition of *sdh2Δ* mutant yeast vs. WT and an irrelevant *nhp6AΔ* gene disruption strain carrying the same *NatR* selectable marker as the *sdh2Δ* strain.

According to the Su accumulation hypothesis [18], [23], [26], [28], [29], SDH mutations inactivate SDH activity and Su accumulates in the cell (Fig. 1A). Su inhibits the 2KG-dependent PHD dioxygenase enzymes that use O_2_ as a substrate to hydroxylate HIFα prolines in normoxia. Because these dioxygenases generate Su, they are susceptible to poisoning by elevated Su concentrations. Thus, SDH loss is thought to disable the TCA cycle and inappropriately activate HIF by Su inhibition of PHD enzymes. The resulting “pseudohypoxic” condition is apparently not tumorigenic in most cell types. In contrast, chronic pseudohypoxic signaling might be a mitogenic stimulus in neuroendocrine cells because these cells may proliferate to mount a futile hormonal response to the perceived hypoxia. Thus, inappropriate HIF persistence due to loss of SDH function in PGL may drive tumorigenesis and HIF is therefore a target for therapy of PGL.

As shown in Fig. 1A, we hypothesize that tumorigenic effects of Su accumulation extend beyond HIF activation by prolyl hydroxylation inhibition [30], to include inhibition of both histone demethylation by Jumoni domain (JHDM) enzymes [31], and 5-methylcytosine hydroxylation by TET1 [32]. Su accumulation could therefore alter gene expression through both altered transcription factor stability and epigenetic effects.

### Rationale for High-throughput Screening in SDH Mutant Yeast Model

The unique features of PGL cells (Su accumulation, loss of TCA cycle, pseudohypoxia, epigenetic changes) raise the possibility that such cells are in a state of stress that could make them differentially susceptible to growth inhibition by small molecules. With no effective cure for the ∼20–60% of metastatic PGLs [6], [33]–[36], small molecules that leverage the metabolic differences between normal and SDH-deficient cells could selectively inhibit the growth of PGL cells and would have immediate therapeutic value. To explore this possibility, our laboratory is developing cell culture and animal models of PGL. Here we report initial results of a high-throughput screen (HTS) using a convenient PGL model provided by a haploid *Saccharomyces cerevisiae* yeast strain carrying an SDH disruption (*sdh2Δ*, corresponding to loss of the mammalian *SDHB* gene). We have previously studied a similar yeast strain as a PGL model [18], and reported correlates with human PGL including the first evidence that Su accumulation can poison Jumoni domain histone demethylases [18].

Yeast cell-based HTS assays have previously been used to successfully identify inhibitors of the HIV-1 HCMV protease [37], membrane bound receptor tyrosine kinases [38], and protein-protein interactions [39]. The eukaryotic context, simple handling, and genetic tractability of budding yeast permit screening for growth inhibitory compounds that are membrane permeable, not generally cytotoxic, and active against proteins in a native context. In this work we create and utilize a haploid *S. cerevisiae sdh2Δ* strain as a PGL model in a HTS for compounds that selectively inhibit the growth of this PGL-like *sdh2Δ* model strain compared to “wild type” (WT) yeast.

## Materials and Methods

### Yeast Strains

Yeast strain genotypes are given in Table 1. The WT strain was obtained from the whole genome knockout collection [40], [41]. This strain carries a disruption of the gene encoding the *PDR5* ABC drug efflux pump, generated by homologous recombination with DNA fragments carrying the G418 resistance marker. Gene disruption by homologous recombination with DNA fragments encoding resistance to clonNAT was used to create the *sdh2Δ*, and *nhp6aΔ* strains. The MX4-NatR gene was amplified with PCR primers conferring 55 bp homology to either *SDH2* (primers: LJM-3547, LJM-3548) or *NHP6A* (primers: LJM-4311, LJM-4312) loci appended to the drug resistance gene from the pFA6-natR-mx4 plasmid [42]. The resulting PCR products were used in a standard lithium acetate yeast transformation, plating transformants on media containing clonNAT. Colony PCR with one primer internal to *NatR* (LJM-4338) and another in the *SDH* or *NHP6A* (LJM-4339) coding region was used to identify colonies with correctly integrated *NatR* inserts (supplemental Fig. S1). The *sdh2Δ adh1Δ* strain was prepared by mating *sdh2Δ* and *adh1Δ* cells of opposite mating types and sporulation of diploids followed by tetrad dissection and selection for spores that gave rise to colonies resistant to both G418 and clonNAT. Rescue of Sdh2 expression in *SDH2Δ* yeast was accomplished by transformation with pJ1528, a pRS426 (*URA3*, 2µ) shuttle vector derivative carrying the *SDH2* gene with 1000 bp upstream sequence.

**Table 1:** *Saccharomyces cerevisiae* strains used in this study.

Strain	Genotype	strain ID
WT	(*MATa his3Δ1 leu1Δ0 met15Δ0 ura3Δ0 pdr5Δ::KanR*)	YL410
*sdh2*Δ	(*MATa his3Δ1 leu1Δ0 met15Δ0 ura3Δ0 pdr5Δ::KanR sdh2Δ::NatR*)	YL411
*nhp6A*Δ	(*MATa his3Δ1 leu1Δ0 met15Δ0 ura3Δ0 pdr5Δ::KanR nhp6aΔ::NatR*)	YL427

### Yeast Growth Media

Yeast media containing different carbon sources were created as follows. 10 g Bacto yeast extract and 20 g Bacto peptone were dissolved in a final volume of 950 mL water. The mixture was autoclaved for 15 min at 121°C followed by the addition of either 50 mL 40% (w/v) galactose or glucose (dextrose) or 50 mL 40% (v/v) glycerol, resulting in YPGal, YPD or YPGly media, respectively.

### High-throughput Screening

For both the LOPAC and 200,000 compound library screens the following process was used. Compound plates were prepared by dispensing 10 µL growth media (YPGal/Gly media) into sterile plates using a Matrix Wellmate instrument. The plates received test compounds using an ECHO550 contactless acoustic nanoliter dispensing system, with the first two and last two columns reserved for no-compound controls. This system dispenses 2.5 nL droplets of 100% DMSO solution containing the dissolved compounds at 10 mM concentration. Twenty droplets were dispensed into the 10 µL of the previously dispensed growth medium to provide a 5× concentration of compound (50 µM). The plates were relidded and transferred back to the Matrix Wellmate where an additional 30 µL of growth media were added to the plates, which were again relidded. This procedure results in a DMSO concentration of 0.125% which was diluted to a final assay concentration to 0.1% DMSO. The *sdh2Δ* strain was grown in YPD medium to provide a stock inoculum of cells for plating. Cells were grown until mid-log phase (approximately 0.8–1.2 OD at 600 nm), harvested by centrifugation, and re-suspended in YPD medium at a cell density of 5×10^6^ cells/mL. 10 µL aliquots of the culture were then dispensed to each well of compound plates using a Beckman FX instrument, resulting in 50 µL cultures containing 1000 yeast cells/µL, 10 µM of compound, and 0.1% DMSO. Cultures were grown in a 30°C humidified incubator and the absorbance at 600 nm was read at 16 h and 48 h.

For each compound in the library, percent inhibition was calculated with the formula:
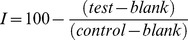
(1)where *test* is the 600 nm absorbance of the well containing 10 µM of compound, *control* is the average 600 nm absorbance of the wells containing yeast culture and no compound, and *blank* is the absorbance of wells containing media alone.

### High-resolution Yeast Growth Curves in 96-well Plates

To compare effects of screen compounds on various yeast strains, YPD yeast cultures were seeded from single colonies and grown overnight to saturation. The resulting cultures were diluted to approximately 1000 cells/µL and grown until log-phase was reached (absorbance of 0.6–0.9 at 600 nm). The log-phase culture cells were collected by centrifugation and washed twice with sterile water to remove the YPD media. The resulting pellet of cells was resuspended in the appropriate liquid media for the experiment. Cultures of 150 µL (1000 cells/µL) were deposited into wells of a 96-well round-bottom polystyrene plate. The plate was lidded and incubated at room temperature with continuous shaking and automated recording of the absorbance (600 nm) every six min.

### Yeast Growth Curve Digitization

Analysis of the high-resolution growth curves was performed with a web-based annotation system that allows user input to guide a curve fitting algorithm based on the work of Toussaint et al. [43]. The blinded fitting procedure identifies the maximal growth rate, lag time, and maximal saturation for each growth phase present (supplemental Fig. S2). The parameters extracted from each fit are stored in a database that can be downloaded as a Microsoft Excel file for statistical testing of hypotheses about differential drug effects on growth.

### In vitro Assays of Yeast Alcohol Dehydrogenase Inhibition

*Saccharomyces cerevisiae* alcohol dehydrogenase, acetaldehyde, and NADH were purchased from Sigma. An automated enzyme assay recorded the absorbance at 340 nm at 2-min intervals over 20 min to measure the enzyme (40 mU/mL)-and NADH (200 µM)-dependent reduction of acetaldehyde (300 µM) in the presence of Tris-HCl, pH 9 (10 mM), ZnCl_2_ (10 nM) and PBS buffer. To assay enzyme inhibition, an 8-point, five-fold serial dilution was completed, yielding final test compound concentrations between 62.5 µM and 0.8 nM. Four replications were completed for each dilution of each compound, and the enzyme inhibition assay was performed three times. 4-methylpyrazole hydrochloride (Sigma) served as a positive control alcohol dehydrogenase inhibitor. Initial reaction rates were plotted as a function of compound concentration, and IC_50_ values estimated using GraphPad Prism.

### Stable Lentiviral SDHB Knockdown in Mammalian Cells

Lentiviral knockdown was performed essentially as described [44]. The human SDHB target sequences were as follows: pJ1824: 5′-A_2_GT_2_GACTCTACT_3_GAC_2_T and pJ1825: 5′-A_3_TCTAC_3_TCT_2_C_2_ACACA-3′ target two independent regions of the SDHB coding sequence. A scrambled sequence (5′- ACTGC_2_GT_2_GT_2_ATAG_2_TG) served as control. Cells were sorted by fluorescence-activated cell sorting for the top 10% mCherry protein expression. Western blotting was used to confirm SDHB knockdown. Antibodies were specific for β-actin (Sigma-Aldrich, A2066) or SDHB (Invitrogen, 459230). Secondary antibodies were horseradish peroxidase conjugated anti-rabbit or anti-mouse antibodies (GE Healthcare, NA934V and NA931V).

### Oxamate Treatment of Mammalian Cells

10^5^ HEK293 cells (infected with the indicated knockdown lentivirus) were seeded in triplicate in a 6-well plate in DMEM medium (Gibco) containing 10% FBS and 1% penicillin/streptomycin for 48 h in the absence or presence of 10 mM oxamate (Sigma). Cells were trypsinized, stained with trypan blue, and live cells counted using a hemocytometer and inverted light microscope.

## Results and Discussion

### Yeast Strain Construction

Three yeast strains (Table 1 and Fig. 1B) were created for the purpose of identifying compounds that act as selective growth inhibitors of *sdh2Δ* mutant yeast. The “wild-type” (WT) yeast strain is derived from BY4741 (*MATa*) with the *PDR5* gene (encoding the ABC drug efflux pump) gene disrupted by homologous recombination with DNA fragments carrying a G418 resistance marker. The choice to use the *pdr5Δ* background was made in order to reduce small molecule efflux by the ABC pump, hence improving the potential for drug accumulation. Based on this WT strain the *sdh2Δ* screening strain and the *nhp6AΔ* control strain were created by disrupting the *SDH2* gene or *NHP6A* gene, respectively, using homologous recombination with DNA fragments encoding a clonNAT resistance marker (see methods). Because the *NHP6A* gene is functionally redundant with the *NHP6B* gene, the *nhp6AΔ* control strain provides a WT-like strain containing the clonNAT resistance marker. This strain combination provided yeast models of both normal and PGL cells, as well as a control with a WT phenotype but bearing the same selectable markers as the *sdh2Δ* strain. This allowed screening for compounds that differentially inhibit growth due to the absence of the *SDH2* gene, and confirmation that the clonNAT resistance marker was uninvolved.

### Yeast Strain Phenotype Validation by Growth on Different Carbon Source

As presumed to be the case for SDH-null PGL tumors, the *sdh2Δ* yeast strain must rely on glycolysis for ATP generation. In contrast, the WT and *nhp6aΔ* yeast strains have intact TCA cycles and therefore have the ability to utilize both glycolysis and oxidative phosphorylation. These predicted phenotypes were validated by growth assays in media containing galactose, glucose (dextrose), or glycerol. Galactose and glucose are fermentable, supporting both glycolysis and oxidative phosphorylation. In contrast, glycerol cannot be fermented and yields energy only by oxidative phosphorylation. The expected phenotypes were confirmed (supplemental Fig. S3A–C). Serial dilutions of the three strains were plated across agar plates containing yeast-extract peptone dextrose (YPD), yeast-extract peptone glycerol (YPGly), or YPD plus 50 µg/mL clonNAT. The observed growth pattern confirms the genotypes, with WT and *nhp6aΔ* strains growing on both YPD and YPGly media and the *sdh2Δ* strain growing only on YPD media. Further, both *sdh2Δ* and *nhp6aΔ* are resistant to clonNAT, with the WT strain remaining sensitive.

**Figure 2:**
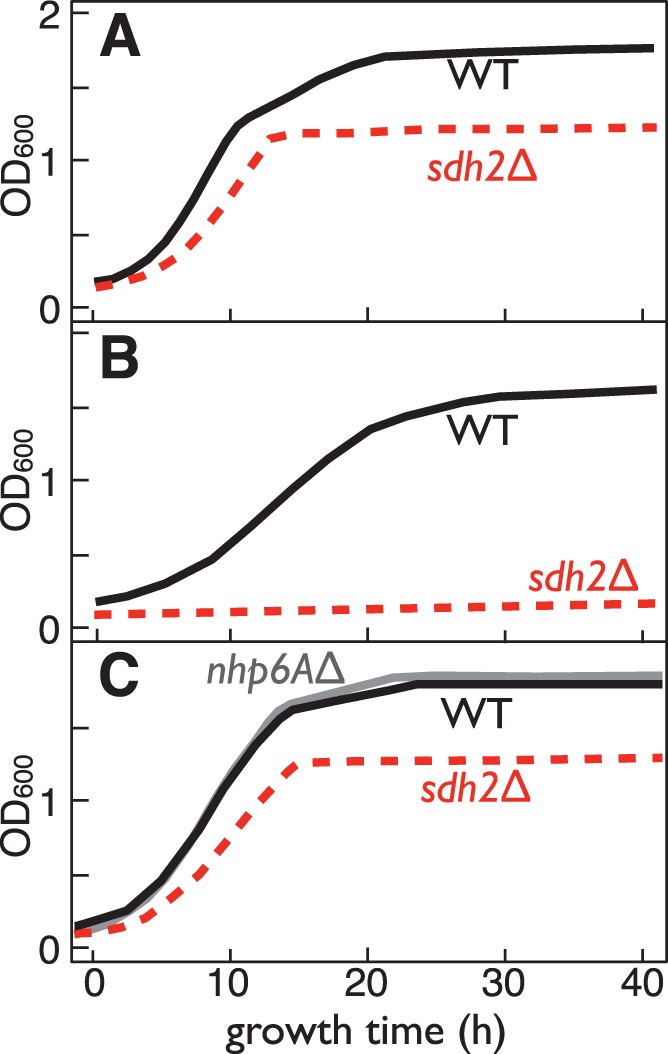
Experimental yeast growth curves on (A) galactose medium; (B) glycerol medium; (C) 1∶1 galactose:glycerol medium. WT (solid black), *sdh2Δ* (dashed red). *nhp6AΔ*, (solid grey).

These metabolic phenotypes were further confirmed in high-resolution growth assays performed in microtiter plates with shaking at room temperature (Fig. 2; supplemental Fig. S3D–F). Yeast cultures were seeded to initial concentrations of 1000 cells/µL in various mixtures of fermentable yeast-extract peptone galactose (YPGal) and non-fermentable YPGly, at ratios of 100∶0, 75∶25, 50∶50, 25∶75, or 0∶100. The absorbance at 600 nm was recorded every six min. Growth curves for the WT and *nhp6aΔ* control strains show clear diauxic shifts upon consumption of all the galactose in the media, and begin to grow by metabolism of the resulting pyruvate derived from glycolysis. As predicted, at just the time that WT and *nhp6aΔ* strains undergo a diauxic shift, the *sdh2Δ* strain ceases to grow, being unable to engage the TCA cycle (supplemental Fig. S3D–F).

### Optimization of 384 Well Plate Yeast Growth Assay

Growth of the WT and *sdh2Δ* yeast strains was compared in a 384-well format appropriate for HTS assays. Every other column of a 384 well plate was seeded with 50 µL cultures of WT or *sdh2Δ* yeast strains in a 50∶50 mixture of YPGal and YPGly media at 30°C. Culture growth was monitored every 2 h for 24 h. In contrast to yeast growth in 96-well format, 50 µL yeast cultures grown in 384-well plates showed evidence of hypoxia. This was evident from the absence of a diauxic shift in WT cultures, leading to similar growth profiles for WT and *sdh2Δ* strains (supplemental Fig. S4). Even with more vigorous shaking and a gas-permeable lid the growth of the WT strain in YPGly media reached only 30% of the total growth reached in two days in the 96-well growth format, indicative of anaerobic growth conditions. This limitation led to a two-step HTS protocol where compounds were first selected for their ability to inhibit growth of the *sdh2Δ* strain under hypoxic conditions in the 384-well plate assay and then retested for selective inhibition of *sdh2Δ* vs. WT strains in a 96 well format with continuous vigorous and adequate oxygenation for aerobic growth.

### Validation of Yeast HTS with the LOPAC Drug Library

An initial screen for compounds that inhibited growth of the *sdh2Δ* strain was performed in triplicate using the Library of Pharmacologically Active Compounds (LOPAC). This library consists of 1280 compounds representing 65 different pharmacological classes (supplemental Table S1). Absorbance measurements were taken at 16 h and 48 h. Several compounds were identified as inhibitors of *sdh2Δ* growth (supplemental Table S2). Each compound in supplemental Table S2 was then retested with high resolution growth curve assays using the *sdh2Δ* and WT strains. Two of these compounds, tetraethylthiuram disulfide (disulfiram), and dequalinium were found to be differentially toxic to the *sdh2Δ* strain (see below).

This initial screen served multiple purposes. First, it validated the biology of the target by identifying two compounds that differentially inhibited growth of the *sdh2Δ* strain. Dequalinium is known to increase ROS levels [45], [46]. Since the *sdh2Δ* strain has previously been shown to suffer from increased ROS levels relative to the WT strain [18] the differential toxicity of dequalinium might reflect the biology of the screen. Similarly, disulfiram had been shown originally to inhibit the enzyme acetaldehyde dehydrogenase [47], [48]. The drug has also been shown to inhibit alcohol dehydrogenase ([49], [50].) Under anaerobic conditions in yeast, alcohol dehydrogenase is required to reduce acetaldehyde to ethanol, regenerating NAD^+^ from the NADH produced by glycolysis. If disulfiram inhibits fungal alcohol dehydrogenase in cells that do not have a functional electron transport chain or are in anaerobic conditions, the cells may deplete NAD^+^ leading to a growth defect. Thus, disulfiram acts as a glycolysis inhibitor and is toxic under anaerobic conditions. The actions of these two compounds thus appear to validate the biological targets of the screen.

The second utility of the LOPAC screen was as a tool to validate the reproducibility and robustness of the screen. This was quantified by calculating the value of the screening *z*-factor and insuring it is within the acceptable limits for HTS. To calculate *z*-factors a positive control is needed. We used the two of the top inhibitory compounds (calmidazolium and ketoconazole, both known broad-spectrum fungicides) from the LOPAC screen to serve as positive controls to calculate z-factors:
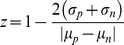
(2)where µ and σ are the mean and standard deviation values of the percent inhibition (equation 1) of the positive (*p*) and negative (*n*) controls, respectively. To obtain μ and σ values, growth plates were created using the screening process for the LOPAC library but with alternating rows of yeast inoculum alone, yeast inoculum and positive control inhibitory compound, or media alone. This assay was performed in quadruplicate to measure assay reproducibility and suitability for HTS. Histograms of the 600 nm absorbance measurements at 16 and 48 h are shown in supplemental Fig. S5 for each of the positive control compounds (ketoconazole and calmidazolium). The z-factors were calculated for each and were greater than 0.5 in all but the 48 h reading of ketoconazole. This might have resulted from ketoconazole degradation or metabolism over time allowing the yeast to escape growth inhibition. For readings at 16 and 48 h the *z*-factors were deemed adequate for HTS.

### HTS Library Screen

Having established proper growth conditions, automated liquid culture handling, yeast growth assay workflow, and yeast strains, we established a HTS of 200,000 additional compounds for selective inhibitors of *sdh2Δ* cells. From the 200,000 compounds screened 175 were identified as inhibiting growth of the *sdh2Δ* yeast strain when the compounds were tested at 10 µM concentration (Table 2). These compounds were then repurchased and assayed at 50 µM for comparative inhibition of WT and *sdh2Δ* cells using a high-resolution growth curve assay. Growth curves were analyzed using a blinded and objective curve annotation system to fit a growth model to each curve in a semi-automated manner (see methods). From each growth curve the maximum culture growth rate, maximum culture saturation, and lag time were each fit for the oxidative and/or glycolytic phases of growth. Any differential effects of the test compounds were quantified by calculating the difference in each of three parameters using the following formulae:
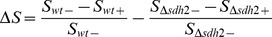
(3)

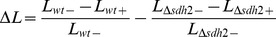
(4)

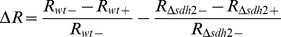
(5)where *S* is the maximum saturation reached by either phase of growth, and *R* and *L* are the growth rate and lag time for the glycolytic phase of growth, respectively. The subscript in all cases represents the strain and the presence or absence of drug. Each of the parameters objectively captures a different differential growth effect that might be exerted by test compounds on the two yeast strains. Similar analyses were performed for initial testing of all repurchased compounds at 50 µM, and for other concentrations subsequently tested.

**Table 2:** HTS results^a^.

Number of compounds	Characteristic
201,200	total compounds initially screened
175	inhibit *sdh2Δ* at 10 µM
14	differential effect on *sdh2Δ* vs. WT
12	*sdh2Δ* inhibited more than WT
2	WT inhibited more than *sdh2Δ*

a 201,200 compounds were screened in two stages, initially identifying 175 compounds that inhibited growth of *sdh2Δ* mutant yeast cells at 10 µM, followed by high-resolution growth curve analysis comparing effects of each compound at 50 µM (and then various lower concentrations) on *sdh2Δ* mutant yeast vs. WT yeast. 14 compounds showed differential growth inhibition, with 12 selectively inhibitory to the *sdh2Δ* mutant yeast. These 12 compounds were not differentially toxic to the *nhp6AΔ* gene disruption strain carrying the same *NatR* selectable marker as the *sdh2Δ* strain.

### Differential Yeast Growth Inhibition

Fourteen compounds were observed to differentially affect the growth of *sdh2Δ* vs. WT yeast at one or more concentrations. Before further analysis, these 14 compounds were tested simultaneously on *sdh2Δ*, WT, and *nhp6aΔ* strains. This control study was undertaken to confirm that drug effects on WT and *nhp6aΔ* were indistinguishable. This was important because the *sdh2Δ* and WT strains actually differ in two ways: *sdh2Δ* lacks a functional *SDH2* gene and it carries the clonNAT resistance marker (encoding an acetyltransferase enzyme). The *nhp6aΔ* control strain carries a WT *SDH2* gene but also clonNAT resistance inserted at an irrelevant genomic site. The observed similarity of drug effects on WT and *nhp6aΔ* yeast rules out that the clonNAT resistance acetyltransferase was unexpectedly enhancing the toxicity of a pro-drug unrelated to loss of SDH activity.

### Compounds that Differentially Inhibit Growth of the WT Yeast Strain

**Figure 3:**
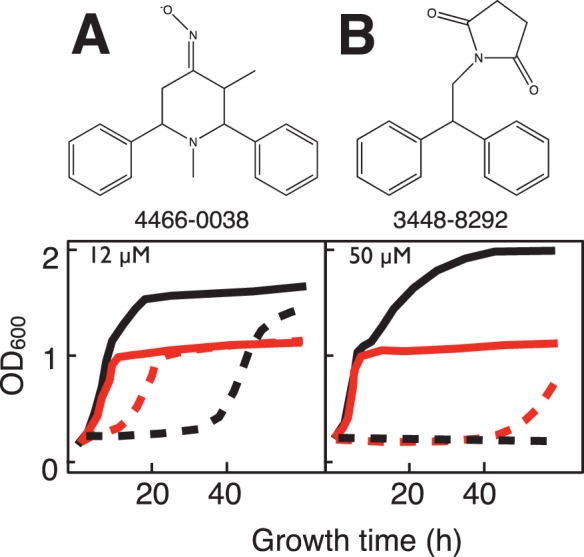
Example growth curves in galactose:glycerol (1∶1) medium showing differential growth inhibition of WT relative to *sdh2Δ* mutant yeast for two compounds. A. 4466-0038 (12 µM). B. 3448-8292 (50 µM). Solid lines: growth in the absence of drug for WT (black) and *sdh2Δ* mutant (red). Dashed lines: growth in the presence of the indicated drug concentration.

Interestingly and surprisingly, two compounds were observed to differentially inhibit growth of WT yeast relative to *sdh2Δ* cells (Fig. 3). The basis for this activity remains unknown, but the clonNAT resistance marker in *sdh2Δ* cells is not implicated because of similar drug effects in *nhp6aΔ* yeast.

### Compounds that Differentially Inhibit Growth of the *sdh2Δ* Yeast Strain

**Figure 4:**
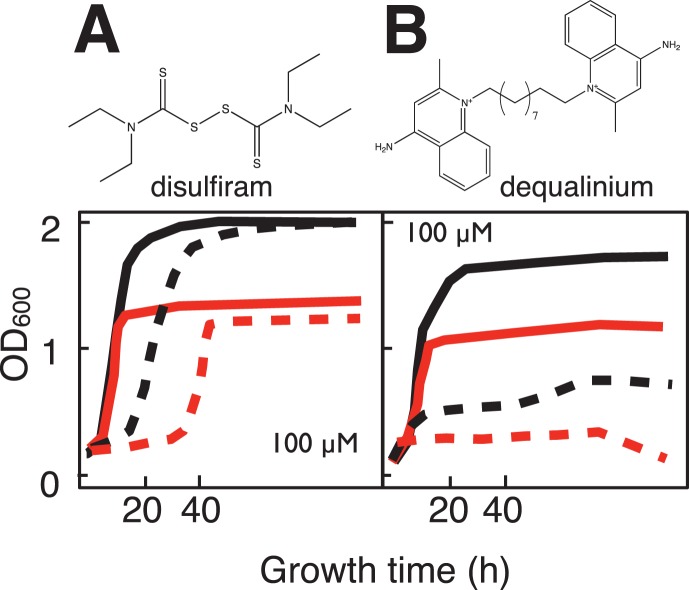
Structures and yeast growth curves in galactose:glycerol (1∶1) medium showing differential growth inhibition of *sdh2Δ* mutant yeast for two compounds from the LOPAC library. A. Disulfiram (100 µM). B. Dequalinium (100 µM). Solid lines: growth in the absence of drug for WT (black) and *sdh2Δ* mutant (red). Dashed lines: growth in the presence of the indicated drug concentration.

**Figure 5:**
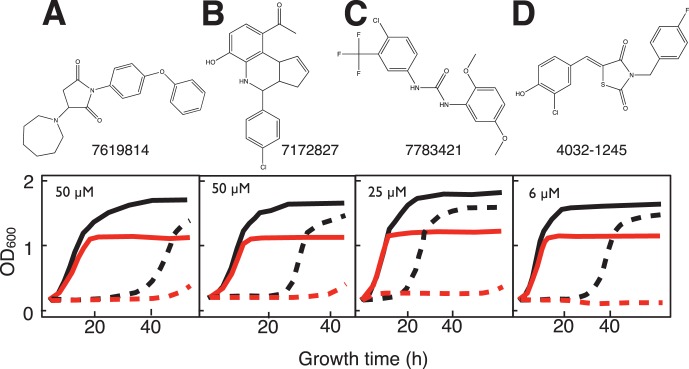
Structures and yeast growth curves in galactose:glycerol (1∶1) medium showing differential growth inhibition of *sdh2Δ* mutant yeast for four non-reactive compounds from main library HTS. A. 7619814 (50 µM). B. 7172827 (50 µM). C. 7783421 (25 µM). D. 4032-1245 (6 µM). Solid lines: growth in the absence of drug for WT (black) and *sdh2Δ* mutant (red). Dashed lines: growth in the presence of the indicated drug concentration.

**Figure 6:**
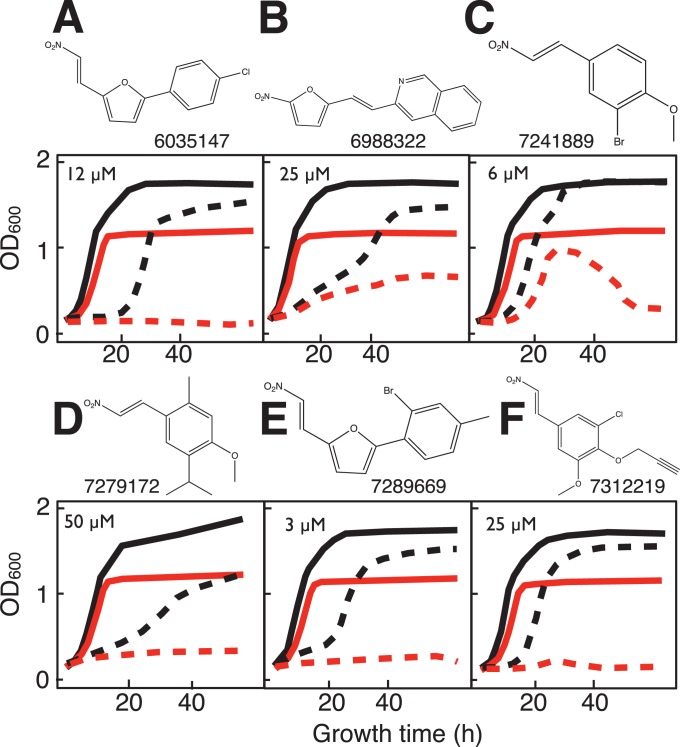
Structures and yeast growth curves in galactose:glycerol (1∶1) medium showing differential growth inhibition of *sdh2Δ* mutant yeast for six nitro-alkene compounds (potential Michael acceptors) from main library HTS. A. 6035147 (12 µM). B. 6988322 (25 µM). C. 7241889 (6 µM). D. 7279172 (50 µM). E. 7289669 (3 µM). F. 7312219 (25 µM). Solid lines: growth in the absence of drug for WT (black) and *sdh2Δ* mutant (red). Dashed lines: growth in the presence of the indicated drug concentration.

Twelve compounds were observed to differentially inhibit growth (saturation, rate or lag time) of the *sdh2Δ* yeast strain (Figs. 4–6). Two of the compounds, disulfiram and dequalinium (Fig. 4) were identified from the preliminary LOPAC screen (described above). Compounds displayed differential toxicity across a range with the most active examples at 3–6 µM and the least active at 100 µM (Table 3). Four compounds (Fig. 5) are not likely to react chemically, while six more (Fig. 6) have activated nitroalkene functions with the potential for covalent chemistry as Michael acceptors.

**Table 3:** Compounds of interest and characteristics.

Identifier	Differentiallyinhibits	Concentration(µM)	IC_50_ WT^a^(µM)	IC_50_ *sdh2Δ* b(µM)	IC_50_ yADH^c^(µM)
7619814	*sdh2Δ*	50	40	25	
7783421	*sdh2Δ*	25			
6035147	*sdh2Δ*	12	35	5	
6988322	*sdh2Δ*	25			
7172827	*sdh2Δ*	50	250	40	25.6
7241889	*sdh2Δ*	6			1.3
7279172	*sdh2Δ*	50			12.7
7289669	*sdh2Δ*	3			
7312219	*sdh2Δ*	25	70	5	3.8
4032-1245	*sdh2Δ*	6			
disulfiram	*sdh2Δ*	100			
dequalinium	*sdh2Δ*	100			
3448-8292	WT	50			
4466-0038	WT	12			

a Compound concentration for 50% growth inhibition of WT yeast strain.

b Compound concentration for 50% growth inhibition of *sdh2Δ* yeast strain.

c Compound concentration for 50% inhibition of yeast alcohol dehydrogenase in vitro.

Four of the 12 selected compounds were tested at multiple concentrations (supplemental Fig. S6) in order to estimate IC_50_ values (Table 3) as examples. For these example compounds, differential sensitivity of the *sdh2Δ* strain in terms of the IC_50_ ratio ranged from 2-fold to 14-fold (Table 3).

**Figure 7:**
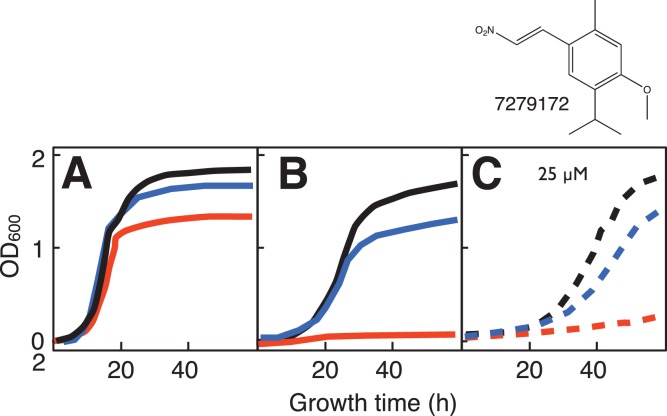
Behavior of representative inhibitor 7279172 on growth of yeast *sdh2Δ pdr5Δ* mutant before and after rescue by plasmid-borne *SDH2*. Growth of WT (black) vs. *sdh2Δ pdr5Δ* mutant (red) vs. *sdh2Δ pdr5Δ* mutant rescued by plasmid-borne *SDH2* (blue) on (A) galactose:glycerol (1∶1) medium; (B) glycerol medium; or (C) galactose:glycerol (1∶1) medium in the presence of 25 µM 7279172.

**Figure 8:**
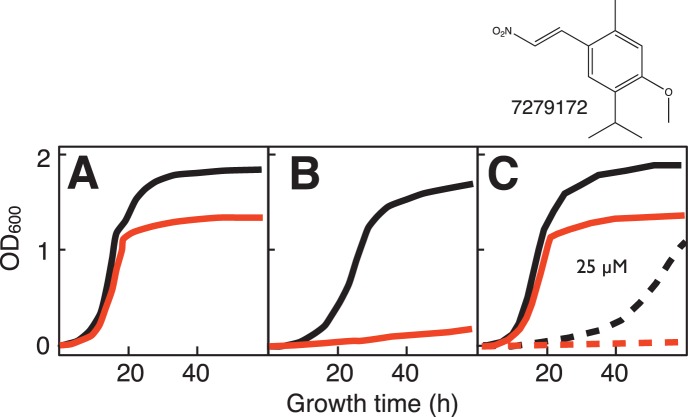
Behavior of representative inhibitor 7279172 on growth of yeast *sdh1Δ* mutant (no *pdr5Δ* mutation). Growth of WT (black) vs. *sdh1Δ* mutant (red) on (A) galactose:glycerol (1∶1) medium; (B) glycerol medium; or (C) galactose:glycerol (1∶1) medium in the absence (solid) or presence (dashed) of 25 µM 7279172, showing differential sensitivity of *sdh1Δ* mutant.

We confirmed the specificity of these results for loss of succinate dehydrogenase activity by performing two control experiments. First, we showed that partial rescue of SDH activity by transformation of the *sdh2Δ pdr5Δ* yeast strain with a plasmid-borne WT copy of *SDH2* conferred resistance to representative growth inhibitor 7279172 (Fig. 7). Second, we showed that differential sensitivity to growth inhibitor 7279172 is not confined to the *sdh2Δ pdr5Δ* screening yeast strain, but is also observed for a *sdh1Δ* mutant yeast strain lacking the *pdr5Δ* mutation (Fig. 8). The latter result shows that disabling the PDR5 efflux pump system is not required for 7279172 activity.

### NAD^+^ Regeneration as a Potential Target for Inhibiting Growth of SDH-deficient Cells

**Figure 9:**
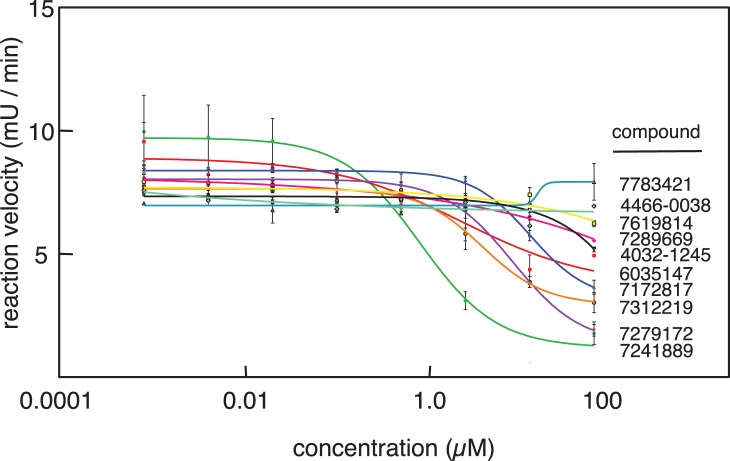
In vitro yeast alcohol dehydrogenase enzyme inhibition assays for ten compounds that differentially inhibit growth of *sdh2Δ* mutant vs. WT yeast.

**Figure 10:**
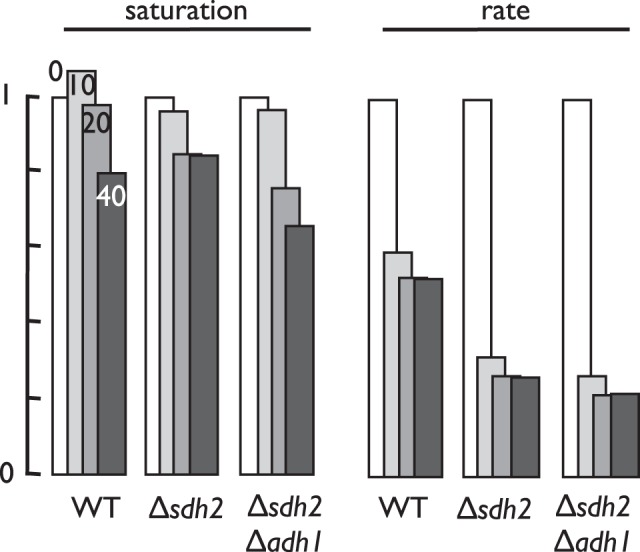
Comparative growth inhibition of WT yeast, *sdh2Δ* mutant yeast, and *sdh2Δ adh1Δ* double mutant yeast by various concentrations (0, 10, 20, 40 µM) compound 7279172 as an example showing sensitization of *sdh2Δ adh1Δ* double mutant yeast to alcohol dehydrogenase inhibitors. Effects of the test compound on maximal culture saturation (left) and time to saturation (right) are normalized to growth in the absence of the compound.

We reasoned that one possible mechanism for differential toxicity to *sdh2Δ* yeast could be inhibition of glycolysis, allowing the TCA-cycle proficient WT yeast to directly metabolize rescuing glycerol in the growth medium. As discussed above with respect to disulfiram, one obvious point of inhibition would be alcohol dehydrogenase, the fungal enzyme required to regenerate NAD^+^ from NADH by reduction of acetaldehyde to ethanol (supplemental Fig. S7). Based on this concept, we developed an in vitro enzyme assay for yeast alcohol dehydrogenase and screened the 10 most promising compounds (7241889, 6035147, 7279172, 7312219, 7172827, 7289669, 7619814, 7783421, 4032-1245, 4466-0038) as alcohol dehydrogenase inhibitors. As shown in Fig. 9, four compounds (7241889, 7279172, 7312219, and 7172817) were indeed found to be potent inhibitors of yeast alcohol dehydrogenase using this in vitro assay. Compounds 7241889, 7312219, and 7279172 were the most impressive inhibitors with average IC_50_ values of 1.3 µM and 3.8 µM, and 12.7 µM, respectively (Table 3). These results validate the notion that alcohol dehydrogenase inhibition is a rational mechanism for selective toxicity to SDH mutant yeast. Besides ADH1, *S. cerevisiae* encodes six other alcohol dehydrogenase enzymes. To confirm the plausibility of alcohol dehydrogenase inhibition as the basis for differential *sdh2Δ* drug sensitivity in vivo, we compared growth inhibition of WT, *sdh2Δ*, and double mutant *sdh2Δ adh1Δ* yeast strains by ADH1-inhibitory compound 7279172 (Fig. 10). These results show that loss of ADH1 increases the sensitivity of *sdh2Δ* yeast to 7279172, as might be expected if the ADH1 enzyme is among the in vivo targets of 7279172.

**Figure 11:**
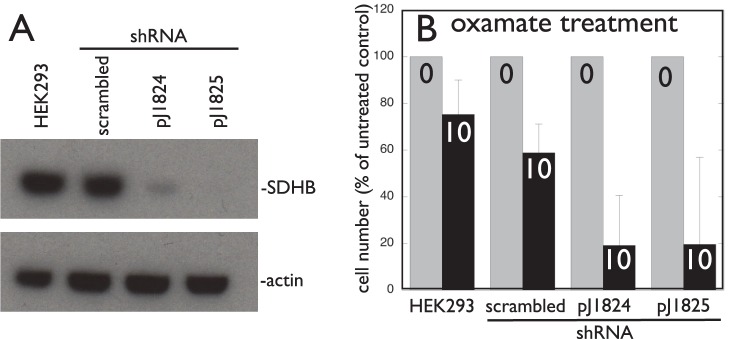
Mammalian cells knocked down for SDHB expression are differentially sensitive to the lactate dehydrogenase inhibitor oxamate. A. Western blot demonstrating stable lentiviral shRNA knockdown of SDHB in HEK293 cells by SDHB-specific shRNA constructs pJ1824 and pJ1825 but not scrambled shRNA. B. Differential 10 mM oxamate inhibition of HEK293 cell growth after SDHB knockdown.

To validate the concept that inhibition of NAD+ regeneration should target SDH-deficient mammalian cells, we performed an experiment using oxamate, a pyruvate analog that selectively inhibits mammalian lactate dehydrogenase [51]. SDHB expression was knocked down by two different stable lentiviral shRNA constructs in human HEK 293 cells (Fig. 11A). The growth of these cells (relative to parental HEK 293 cells or cells transduced with a scrambled shRNA lentivirus) was differentially inhibited by oxamate (Fig. 11B).

## Insights and Discussion

As described above, the detection of approved LOPAC drugs disulfiram and dequalinium among compounds differentially toxic to *sdh2Δ* mutant yeast cells is interesting and provocative. These agents have been proposed as anti-malarials, with disulfiram as a potential inhibitor of alcohol dehydrogenase and dequalinium potentially increasing reactive oxygen species, a stress already known to be higher in *sdh2Δ* mutant yeast [18].

Interestingly, six of the ten compounds identified by the main HTS are potentially chemically reactive as Michael acceptors. Related compounds are sometimes categorized as pan assay interference “PAINS” compounds that can be problematic in HTS by interference with fluorescent detection or other technical problems [52]. However, it must be noted that these compounds were identified here in a simple differential growth screening protocol that avoids nonspecific effects commonly associated with PAINS compounds. In fact, the compounds shown in Fig. 6 reflect potentially reactive agents as have been enjoying a resurgence in medicinal chemistry [53] through covalent mechanisms including thiol-reactivity [54]–[56].

With respect to possible mechanisms of differential toxicity to *sdh2Δ* mutant yeast cells, the most obvious *a priori* targets include the unique reliance of *sdh2Δ* mutant cells on glycolysis and the potentially stressful increases in intracellular ROS and Su [18]. If each of these characteristics holds for SDH mutant human PGL tumor cells, rational approaches might be imagined. In the simplest case, PGL cells could be starved by providing only non-fermentable carbon sources if metabolism in other tissues could be supported by ketogenic agents.

Our HTS screening results add insights into possible clinical tactics against SDH mutant cells such s SDH-mutant PGL. Our observation that alcohol dehydrogenase inhibitors are differentially toxic to *sdh2Δ* mutant yeast is interesting and provocative. Disulfiram is an approved anti-alcoholism medication shown to inhibit both aldehyde dehydrogenase and alcohol dehydrogenase [47]–[50]. Disulfiram is also an anti-malarial, noteworthy because, like PGL cells, *P. falciparum* cells lack a TCA cycle and rely on glycolysis. Moreover, we have now shown that four of the ten leading differential inhibitors of *sdh2Δ* mutant yeast growth are inhibitors of yeast alcohol dehydrogenase. This result points to glycolysis inhibition as an interesting approach in human PGL (supplementary Fig. S7). In the absence of a TCA cycle, NAD^+^ recycling to NADH might become limiting for PGL tumor cells. Yeast uses pyruvate decarboxylase to convert pyruvate to acetaldehyde and then alcohol dehydrogenase to regenerate NAD^+^ by reduction of acetaldehyde to ethanol at the expense of NADH. Mammals accomplish the same NAD^+^ regeneration by reducing pyruvate to lactate using lactate dehydrogenase. Thus, by analogy with the results of this high-throughput screen, an interesting target for therapeutic inhibition of SDH mutant PGL would be lactate dehydrogenase [51]. We confirm this concept here by showing that human cells deficient in SDH activity are differentially sensitive to the lactate dehydrogenase inhibitor oxamate. This provocative result points to the potential value of lactate dehydrogenase inhibitors in paraganglioma therapy.

## Supporting Information

Figure S1 **Test for proper integration of** ***NatR*** **gene into** ***NHP6A*** **gene locus.** A) PCR products for WT, *sdh2Δ*, and *nhp6aΔ* strains resulting from amplification with primers LJM-4338 in the *NHP6A* gene region and LJM-4339 internal to the *NatR* gene insert. The expected ∼600 bp PCR product is observed in the *nhp6aΔ* strain indicating disruption of the *NHP6A* gene with the *NatR* gene insert. B) PCR products for amplification of WT, *sdh2Δ*, and *nhp6aΔ* strain genomic DNA with primers (LJM-4310, LJM-4335) specific for the *NHP6A* gene region. PCR products of the expected size for both the WT and *sdh2Δ* strains indicate the presence of the intact *NHP6A* gene and absence of PCR product for genomic DNA from the *nhp6aΔ* strain indicate *NHP6A* disruption in the latter.
(EPS)

Figure S2 **Schematic illustration of fitting parameters used to extract growth curve characteristics for objective scoring of screened compound effects.** A. Illustration of lag parameter for multiphasic growth curves. B. Illustration of saturation (sat) and lag parameters in the absence (−) and presence (+) of screened compound.
(EPS)

Figure S3 **Verification of yeast strains.** A. Serial dilutions across agar plate containing the fermentable carbon source, dextrose (glucose). B. Serial dilutions across agar plate containing non-fermentable carbon source, glycerol. C. Serial dilutions across agar plate containing glucose and clonNat. D–F. Growth curves of yeast strains in liquid media containing the indicated ratios of fermentable and non-fermentable carbon sources: D, WT; E, *sdh2Δ*; F, *nhp6aΔ*.
(EPS)

Figure S4 **Yeast growth assay in 384 well plate.** WT (solid) or *sdh2Δ* (open) yeast strains were grown in 50 µL cultures in 384 well plates. The plot represents the distribution of the absorbance at 600 nm for each yeast strain grown in YPGal (red) or a 50∶50 mixture of YPGal and YPGly media (black).
(EPS)

Figure S5 **Histograms of yeast growth for control plates used to calculate z-factors for HTS.** A) Histogram plots of 16 and 48 h OD_600_ readings for a single 384 well plate ordered by row along the x-axis. Each row alternates between *sdh2Δ* (red), *sdh2Δ* plus calmidazolium (green), and media alone (blue). The 48 h readings maximize the differences between *sdh2Δ* and *sdh2Δ* plus calmidazolium while minimizing the variance within each group for calmidazolium, and are optimal for HTS B) Similar histograms generated from control plates using ketoconazole show optimal conditions at 16 h.
(EPS)

Figure S6 **Concentration-dependent growth inhibition curves for example compounds 6035147, 7172827, 7312219, 7619814.** Maximal yeast growth is shown as a function of compound concentration to allow estimation of the compound concentration necessary for 50% growth inhibition (IC_50_) as shown in Table 3.
(EPS)

Figure S7 **Anaerobic regeneration of NAD^+^ in yeast vs. human cells.** Fungal alcohol dehydrogenase regenerates NAD^+^ by NADH-dependent reduction of acetaldehyde, whereas NAD^+^ regeneration in mammals is by NADH-dependent reduction of pyruvate by lactate dehydrogenase. Differential toxicity of alcohol dehydrogenase inhibitors for *sdh2Δ* mutant yeast suggests that lactate dehydrogenase inhibitors might be differentially toxic for SDH mutant human PGL tumors.
(EPS)

Table S1 **Compound classes in LOPAC 1280 library.**
(DOC)

Table S2 **LOPAC 1280 compounds that significantly inhibit growth of *sdh2Δ* mutant yeast.**
(DOC)
