# Supplementary material for: High-Throughput Screening for Growth Inhibitors Using a Yeast Model of Familial Paraganglioma
Source: PLoS One. 2013 Feb 22;8(2):e56827. doi: 10.1371/journal.pone.0056827 (PMC3579935; doi:10.1371/journal.pone.0056827)
Supplement: Table S1 — Compound classes in LOPAC 1280 library. (DOC) [file pone.0056827.s008.doc]

Table S1. Compound classes in LOPAC 1280 library

| Class | Number of drugs | Class | Number of drugs |
| --- | --- | --- | --- |
| Adenosine | 53 | Hormone | 36 |
| Adrenergic | 1 | Imidazoline | 10 |
| Adrenoceptor | 103 | Immune System | 11 |
| Angiogenesis | 1 | Inflammation | 1 |
| Antibiotic | 28 | Intracellular Calcium | 7 |
| Anticonvulsant | 11 | Ion Channels | 1 |
| Apoptosis | 14 | Ion Pump | 16 |
| Benzodiazepine | 6 | K^+^ Channels | 22 |
| Benzodiazepine | 1 | Leukotriene | 10 |
| Biochemistry | 44 | Lipid | 9 |
| Ca^2+^ Channel | 19 | Lipid Signaling | 2 |
| Calcium Signaling | 1 | Melatonin | 7 |
| Cannabinoid | 5 | Multi-Drug Resistance | 12 |
| Cell Cycle | 16 | Na^+^ Channel | 19 |
| Cell Stress | 18 | Neurodegeneration | 2 |
| Cholecystokinin | 3 | Neurotransmission | 48 |
| Cholinergic | 79 | Nitric Oxide | 34 |
| Cl^-^ Channel | 3 | Nootropic | 3 |
| Cyclic Nucleotides | 31 | Opioid | 27 |
| Cytokines & Growth Factors | 1 | P2 Receptor | 14 |
| Cytoskeleton and ECM | 10 | Phosphodiesterase | 3 |
| DNA | 9 | Phosphorylation | 97 |
| DNA Metabolism | 13 | Prostaglandin | 23 |
| DNA Repair | 3 | Serotonin | 88 |
| Dopamine | 107 | Sigma receptor | 1 |
| G protein | 3 | Somatostatin | 1 |
| GABA | 41 | Sphingolipid | 4 |
| Gene Regulation | 2 | Tachykinin | 5 |
| Glutamate | 90 | Thromboxane | 1 |
| Glycine | 2 | Transcription | 12 |
| Histamine | 31 | Vanilloid | 5 |
