# Supplementary material for: High-Throughput Screening for Growth Inhibitors Using a Yeast Model of Familial Paraganglioma
Source: PLoS One. 2013 Feb 22;8(2):e56827. doi: 10.1371/journal.pone.0056827 (PMC3579935; doi:10.1371/journal.pone.0056827)
Supplement: Table S2 — LOPAC 1280 compounds that significantly inhibit growth of sdh2Δ mutant yeast. (DOC) [file pone.0056827.s009.doc]

Table S2. LOPAC 1280 compounds that significantly inhibit growth of *sdh2Δ* mutant yeast.
